# Rituximab induced hypoglycemia in non-Hodgkin's lymphoma

**DOI:** 10.1186/1477-7819-4-89

**Published:** 2006-12-11

**Authors:** Badrudeen M Hussain, N Geetha, V Lali, Manoj Pandey

**Affiliations:** 1 Division of Medical Oncology, Regional Cancer Center, Trivandrum, Kerala, India; 2 Division of Surgical Oncology, Regional Cancer Centre, Thiruvananthapuram, Kerala, India; 3 Department of Surgical Oncology, Institute of Medical Sciences, Banaras Hidnu University, Varanasi 221 005, India

## Abstract

**Background:**

Hypoglycemia is a vary rare toxicity of rituximab. The exact mechanism of rituximab induced hypoglycemia is not clear.

**Case presentation:**

A 50 year old female presented with a left tonsillar non Hodgkin's lymphoma and was started on R-CHOP chemotherapy. Twenty four hours after the first rituximab infusion, she developed hypoglycemia which was managed by IV glucose infusion.

**Conclusion:**

Hypoglycemia following rituximab administration is rare. Possibilities of hypoglycemia should be kept in mind in patients developing symptoms like fatigue, restlessness, and sweating while on rituximab therapy.

## Background

Hypoglycemia is a rare complication of rituximab treatment. The exact cause of this phenomenon is not known. We report here a case of non-Hodgkin's lymphoma developin hypoglycemia on being treated with R-CHOP chemotherapy

## Case presentation

A 50-year-old female presented in January 2004 with a 4 cm left tonsillar mass. Her general and systemic examinations were unremarkable. There was no lymphadenopathy or hepato-splenomegaly. Her hemoglobin was 12.5 gm/dl, white blood cell (WBC) count was 7700/mm^3^, erythrocyte sedimentation rate (ESR) was 50 mm/hour and random blood sugar (RBS) was 96 mg/dl. Renal and liver function tests were normal. VDRL, HIV and HBsAg were non-reactive. Chest X-ray, ultrasound scan of the abdomen, upper gastro-intestinal endoscopy, peripheral blood smear and bone marrow biopsy were normal. There was no past history of diabetes mellitus, hypo or hyperglycemic episodes. Biopsy and histopathological examination of the tonsillar mass was consistent with Non Hodgkin's lymphoma-diffuse large B cell type. Immunohistochemistry showed the tumor cells to be CD20 positive. She was treated using R-CHOP (rituximab 375 mg/m^2 ^+ cyclophosphamide 750 mg/m^2 ^+ doxorubicin 50 mg/m^2 ^+ vincristine 1.4 mg/m^2 ^+ prednisolone 100 mg orally) chemotherapy on a three weekly schedule after obtaining informed consent. About 24 hours following rituximab, she developed hypoglycemic symptoms like fatigue, restlessness, sweating and drowsiness. There was no other rituximab infusion related toxicities. Her RBS at that time was 39 mg/dl. Her symptoms subsided following intravenous (IV) glucose administration and RBS became normal. During the next 48 hours, she developed three more symptomatic hypoglycemic episodes, with RBS nadir 54 mg/dl, 61 mg/dl and 70 mg/dl respectively and were managed with IV glucose administration. Three days after rituximab her hypoglycemic symptoms subsided completely and the RBS improved to normal range. During the subsequent courses of rituximab, she was given IV glucose support during the first 72 hours. There were no further episodes of symptomatic hypoglycemia.

## Discussion

Rituximab is a chimeric murine/human monoclonal antibody directed against CD-20 positive cells. Commonly observed toxicities are fever(55%), chills(33%), asthenia(25%), lymphopenia (48%), nausea (23%), bronchospasm (10%), anemia (15%), neutropenia (15%), abdominal pain (14%), back pain (10%), flushing (5%), angio-edema (11%), peripheral edema (8%), night sweats (15%) hypotension (10%) and hypertension (6%). Metabolic complications like hyperkalemia, hyperuricemia, hyperphosphatemia and hypocalcemia have been observed 12–24 hours after an infusion due to an association with a reduction in tumor burden. Rarely hyperglycemia is also seen [1,2]. But occurrence of hypoglycemia is vary rare and is reported in more than 1 but less than 5% of patients. The exact mechanism of rituximab induced hypoglycemia is not clear [2]. Rituximab has also been used to treat type B syndrome of severe insulin resistance in one patient earlier [3].

## Conclusion

Even though its occurrence is rare, the possibility of hypoglymia should always be kept in mind.

## Conflict of interest

The author(s) declare that they have no competing interests.

## Authors' contributions

BMH literature review and preparation of draft manuscript

NG and VL helped in preparation of manuscript

MP helped in preparing the draft manuscript and edited the final version.
